# Maternal iron status during pregnancy and attention deficit/hyperactivity disorder symptoms in 7-year-old children: a prospective cohort study

**DOI:** 10.1038/s41598-022-23432-1

**Published:** 2022-12-01

**Authors:** Andrés Díaz-López, Josefa Canals-Sans, Jordi Julvez, Silvia Fernandez-Barrés, Sabrina Llop, Marisa Rebagliato, Nerea Lertxundi, Loreto Santa-Marina, Mònica Guxens, Jordi Sunyer, Victoria Arija

**Affiliations:** 1 Nutrition and Mental Health Research Group (NUTRISAM), Rovira I Virgili University (URV), C/ Sant Llorenç 21, 43201 Reus, Tarragona Spain; 2 Institut d’Investigació Sanitària Pere Virgili (IISPV), Tarragona, Spain; 3 Research Center for Behavioral Assessment (CRAMC), Rovira I Virgili University (URV), Tarragona, Spain; 4 Barcelona Institute for Global Health (ISGlobal), Barcelona, Spain; 5 Epidemiology and Environmental Health Joint Research Unit, FISABIO-Public Health, FISABIO-Universitat Jaume I-Universitat de València, Valencia, Spain; 6 Spanish Consortium for Research On Epidemiology and Public Health (CIBERESP), Instituto de Salud Carlos III, Madrid, Spain; 7 Biodonostia, Epidemiology and Public Health Area, Environmental Epidemiology and Child Development Group, San Sebastian, Spain; 8 Faculty of Psychology, University of the Basque Country (UPV/EHU), San Sebastian, Spain; 9 Pompeu Fabra University, Barcelona, Spain; 10 Department of Child and Adolescent Psychiatry/Psychology, Erasmus MC, University Medical Centre, Rotterdam, The Netherlands

**Keywords:** Cognitive neuroscience, Predictive markers, Nutrition, Epidemiology, Neurological disorders

## Abstract

Evidence suggests that iron status may be linked to symptoms of childhood attention deficit/hyperactivity disorder (ADHD), but there is little data available on the relationship between iron status in pregnancy and the risk of developing ADHD. And the data that does exist is inconsistent. Our aim here is to assess the effect of maternal serum ferritin (SF) and haemoglobin (Hb) levels during pregnancy on manifestations of ADHD in children at 7 years of age. This prospective study analysed data from 1204 mother–child pairs from three Spanish cohorts participating in the INMA project. Maternal SF and Hb levels during pregnancy and other mother and child characteristics were collected. The children’s ADHD behaviours were reported by their parents using Conners’ Parent Rating Scale-Revised Short Form (CPRS-R:S). In the unadjusted regression analysis, maternal SF was positively associated with children’s T-scores on the subscales Cognitive problems/Inattention (β: 0.63, 95%CI 0.06–1.19; *p* = 0.029) and ADHD index (β: 0.72, 95%CI 0.20–1.24; *p* = 0.007). These associations were not present after multivariate adjustment or stratification by first and second trimester of pregnancy. The Hb levels were not related to any of the CPRS-R:S subscales in unadjusted or multivariate-adjusted models. We observed no association between maternal SF or Hb levels and the risk of ADHD symptomatology (T-score ≥ 65 for CPRS-R:S subscales). Our results suggest that neither maternal SF nor Hb levels during pregnancy are related to ADHD symptoms in 7-year-old children.

## Introduction

Attention-deficit/hyperactivity disorder (ADHD) is the most common neurodevelopmental disorder, with a worldwide prevalence of 5–7.7% among school-aged children^1,2^. It is characterised by a constellation of symptoms that include inattention, impulsivity, hyperactivity, and altered executive functions. The disorder has also been linked to other psychological alterations, such as disruptive behaviours, processing speed problems, reading and writing difficulties, and emotional problems, resulting in impaired learning and adaptative ability in children^3^. The precise aetiology of ADHD remains unclear, but evidence indicates that it is a multifactorial disorder with a strong genetic component (with heritability averaging 76%)^4^ and in which early environmental factors can also play an aetiological role or trigger expressions of different behaviours in genetically predisposed children^5^. Thus, identifying early modifiable determinants of childhood ADHD could help to identify specific actions to maximise the impact on public health.

Some epidemiological studies suggest that nutritional factors^6^ during pregnancy—a critical period for brain development—such as maternal intake of iron^7^, folic acid^8^, omega-3^9^, vitamins D^10^ and B12^11^, and caffeine^12^ may be linked to symptoms of ADHD in childhood. A good prenatal iron status is essential for correct neurodevelopment in children^13^ since it plays a key role in regulating dopaminergic activity, myelination, and other processes related to brain growth and function^14,15^. Low maternal iron levels during pregnancy, assessed by serum ferritin (SF) concentration, can cause iron deficiency in the brain in offspring, leading to neurocognitive disturbances related to neurodevelopmental disorder^15,16^. Unfortunately, neurocognitive alterations caused by iron deficiency during the critical prenatal period of brain development are difficult to remedy and persist later in life^16^. In this regard, low SF levels in the umbilical cord have been associated with poorer auditory recognition memory in newborns^17^ and impaired cognitive and psychomotor development in early infancy^18–20^. However, to our knowledge, only two prospective studies have evaluated prenatal iron status and ADHD in children of different ages, with mixed results^7,21^. In a large cohort of Swedish women and their offspring aged 6–29 years, Wiegersma et al.^21^ found that iron deficiency anaemia during early pregnancy (at ≤ 30 weeks of gestation) was associated with a greater risk of ADHD, co-occurring with autism spectrum disorder and/or intellectual disability disorder. However, associations did not reach statistical significance with anaemia diagnosed later in pregnancy (at > 30 week of gestation) or when only ADHD was considered as the outcome. Along similar lines, in our Spanish INMA (Environment and Childhood) birth cohort^7^ an inverse association between maternal SF in early pregnancy and inattention and total ADHD symptoms in boys at pre-school age (4 years) has been previously reported. However, SF levels were not predictive of hyperactivity/impulsivity symptoms^7^.

The little evidence and the inconsistent findings in this field of research point to a need for further research. Furthermore, it remains to be determined whether the association between prenatal SF and ADHD symptoms persists into school age, when environmental factors that affect children’s development and learning could act as modulators. Likewise, there is evidence to suggest that maternal iron status during pregnancy affects child neurodevelopment differently depending on the trimester of pregnancy^13^. However, little is known about the trimester in which the foetus is most susceptible to prenatal iron status or indeed how this might affect child ADHD symptomatology. Although we previously^7^ reported an inverse association between maternal SF levels and child ADHD symptom scores, it is not known if the scores indicated a clinical diagnosis of ADHD. This relationship may also be confounded by other maternal ADHD-related factors, regardless of genetics, such as sociodemographic and/or lifestyle factors, inflammation, and others. Previous studies have rarely considered a large range of risk factors simultaneously, which increases the possibility of residual confounding.

Considering the body of literature described above, we hypothesised that higher maternal SF and Hb levels during pregnancy act as protective factors for the development of ADHD symptoms at school age. As an additional follow-up analysis in the same INMA birth cohort, this study (1) investigates the relationship between maternal SF and Hb levels during pregnancy and manifestations of ADHD symptoms in school-aged children at 7 years old and (2) assesses the relationship between these maternal iron parameters and gestation period.

## Methods

### Study design and population

This longitudinal population-based study analyses data from healthy pregnant women and their children from three sub-cohorts of the INMA project: Valencia (Valencian Community), Sabadell (Catalonia) and Gipuzkoa (Basque Country) (http://www.proyectoinma.org). INMA is an ongoing multicentre mother–child cohort study evaluating the long-term impact of environmental exposures and diet during pregnancy on child growth and development. The study design and protocol are described in detail elsewhere^22,23^. The population-based INMA birth cohort used a common protocol^22^ to recruit 2,150 pregnant women at approximately 12 weeks of gestation (first trimester of pregnancy) from the general population in three Spanish regions between 2003 and 2008. Women were eligible if they met the following inclusion criteria: age ≥ 16 years, ability to communicate in Spanish, intention to deliver at the reference hospital, singleton pregnancy, and no assisted conception. This study was approved by the Clinical Research Ethical Committees of Donostia (Gipuzkoa), La Fe (Valencia) Hospitals, and the Medical Assistance Municipal Institute (Barcelona) and written informed consent was obtained for all participants from parents or legal guardians. The study complies with the tenets of the Declaration of Helsinki.

Women were enrolled during pregnancy and monitored together with their children until the children reached the age of 7–8 years. Mother–child pairs were included in the present study only if they had been measured for maternal SF during pregnancy and child ADHD symptoms with Conners’ Parent Rating Scale-Revised Short Form (CPRS-R:S) at approximately 7 years old. The final study population consisted of 1204 mother–child pairs.

### Assessment of serum ferritin and haemoglobin measurements

The main exposure of interest for this study was maternal iron status during pregnancy, which was assessed in terms of SF concentrations, an established biomarker of body iron. Maternal ferritin (µg/L) levels were measured at a single point in the first trimester (mean 9.6 weeks, standard deviation (SD) 2.5) or second trimester (mean 14.6 weeks, SD 2.3) of pregnancy in serum samples collected and stored at − 80 °C until assessment. SF measurements in the first and second trimester as a whole (combined) were defined as the first period of pregnancy and analysed as total SF levels. For the Gipuzkoa and Sabadell cohorts, SF concentrations were quantified by time-resolved fluorescence immunoassay (DELFIA Ferritin kit A069–101) at the Gipuzkoa Public Health Laboratory, and for the Valencia cohort they were quantified by immunoturbidimetry (Beckman Coulter AU analysers) at La Fe hospital. The secondary exposure of interest was Hb. Data on maternal Hb levels (g/dL) were also obtained during the first trimester (mean 9.6 weeks, standard deviation (SD) 2.7) and third trimester (mean 27.2 weeks, SD 4.2) of pregnancy from medical records. Iron deficiency was defined as SF levels < 12.0 µg/L and anaemia as Hb levels < 11.0 g/dL.

### Assessment of children’s ADHD symptoms

At a median age of 7.5 (IQR, 0.8) years old, child ADHD behaviours were reported by parents using the CPRS-R:S^24^, which consists of 27 items distributed in four subscales: Oppositional (6 items), Cognitive problems/Inattention (6 items), Hyperactivity (6 items), and an ADHD index (12 items). Each item was rated on a 4-point Likert scale (0 ‘not true at all’ to 3 ‘very much true’). For each subscale, raw total scores were summed to provide continuous measures and converted into T-scores based on age-and-sex appropriate reference groups, with higher scores indicating greater severity of ADHD. In the INMA sample, the CPRS-R:S showed good internal consistency for each of the four subscales (α = 0.85, α = 0.89, α = 0.82, and α = 0.91, respectively). To apply a more clinical approach, we also generated dichotomous variables of ADHD risk using a T-score ≥ 65 (elevated score) as the cut-off point on any of the CPRS-R:S scales. This score is indicative of a ‘significant problem’^24^.

It is important to note that all the psychometric assessment tests were reviewed/checked to ensure that they had been correctly completed, by cohort-based psychologists with extensive experience in the INMA Project fieldwork.

### Assessment of covariates

Information on mother and child characteristics was obtained through face-to-face interviews conducted by trained interviewers at different times in the first and third trimester of pregnancy, at birth, and when the children reached 7–8 years of age. Maternal variables included cohort (Gipuzkoa, Valencia, Sabadell), age (years), maternal pre-pregnancy body mass index (BMI) (kg/m^2^), educational level (primary or less, secondary, university), social class (occupation during pregnancy coded according to the International Standard Classification of Occupations-88 system: high status (I–II comprising managers/technicians), medium status (III comprising skilled workers), low status (IV–V comprising semiskilled/unskilled workers), ethnic group (Caucasian, others), smoking during pregnancy (yes, no), alcohol consumption during pregnancy (g/d), mode of delivery (eutocic [spontaneous vaginal delivery without instrumental intervention], dystocic [urgent or elective caesarean section] or instrumental vaginal delivery [vacuum, forceps or spatulas]), parity (primiparous [no previous live birth], multiparous [one or more live births]), and breastfeeding duration (weeks of breastfeeding).

Dietary intake of iron during pregnancy was assessed with a validated 101-item semi-quantitative food frequency questionnaire (FFQ)^25^ collected at 28–32 weeks of gestation (to estimate dietary intakes during second and third trimesters). Reference values from the U.S. Department of Agriculture (USDA) and Spanish food composition tables^26,27^ were used to calculate iron intake (mg/d), which was adjusted for energy intake using the residual method^28^. Data on iron supplement use and dose was collected using a structured questionnaire at the same time (28–32 weeks) as the FFQ. Total daily iron intake was obtained by adding daily iron intake (in mg) from diet and from supplements, and classified as ≤ 27 or > 27 g/d according to the recommended dietary allowances (RDA) for pregnant women.

C-reactive protein (CRP) levels were also measured in serum samples collected during the first trimester in only two cohorts using an immunoturbidimetric method at Consulting Quimico Sanitario Laboratory (Gipuzkoa cohort) and Echevarne Sabadell Laboratory (Sabadell cohort). The CRP level was used to assess the presence of infection or inflammatory conditions and to complement the SF information. In our population of healthy pregnant women, CRP levels ranged from 0.2 to 5.4 mg/L. Values at or above 0.65 mg/L (corresponding to the 75th percentile) were considered to be high.

Information about the children’s sex (male, female), as well as anthropometric measures at birth such as standard weight (grams) and head circumference (cm) at gestational week 40, and gestational age (weeks, calculated from the date of the last menstrual period reported at recruitment and confirmed using ultrasound examination at week 12 of gestation) were obtained from clinical records. The weight and height of each child were measured at 7 years of age, and BMI was used to estimate age- and sex-specific z-scores. The children’s exact age (years) was also obtained at the time of the assessment of ADHD symptoms.

### Statistical analysis

Descriptive data is presented as a number (percentage) for categorical variables and as a mean (± SD) for continuous variables unless otherwise indicated. SF and Hb were assessed for normality using both the Shapiro–Wilk test and visual inspection (quantile–quantile plot). Because of skewed distributions, SF was log transformed before analyses. Comparisons of general characteristics between cohorts and categories of CPRS-R:S scores were assessed by ANOVA, chi-square or Student’s t tests, as appropriate.

For the analyses, maternal SF (log transformed) and Hb concentrations were treated both as continuous variables and categorical variables using tertiles, with the lowest tertile as reference. Associations between each maternal iron parameter (including total SF levels in the first period of pregnancy [first and second trimester combined], and trimester-specific levels of SF [first and second trimester] and Hb [first and third trimester]) and the children’s T-scores on the four CPRS-R:S subscales (as continuous variables) at 7 years old were evaluated using separate univariate and multivariate linear regression analyses. The results are shown as β coefficient and 95% confidence intervals (CIs). Based on previously reported/known associations/risk factors for ADHD symptoms^7^, we selected a priori maternal and child covariates as potential confounders. These are summarized in Table 1 and only those covariates that had a p-value < 0.05 in the bivariate associations for each CPRS-R:S subscale were included in the final multivariate models. Multicollinearity was assessed by inspection of the tolerance (1/VIF) values and variance inflation factors (VIFs). All tolerance values were greater than 0.6 and all VIFs less than 2.0, suggesting no concerns with multicollinearity. We also tested for interactions between maternal SF and Hb levels and all the post-exposure child covariates (i.e. children’s sex, age at delivery, birth weight, birth head circumference, children’s age at the time of the test, and BMI-for-age z-score at 7 years of age) with the likelihood ratio tests, which involved comparing full models with and without interaction terms. In all cases, they were not significant at the significance level of ≤ 0.05. Nevertheless, the covariates were retained in the multivariate models because they were known to be closely related to ADHD symptoms. Tests for trend were conducted using the median values for each tertile of SF or Hb as a continuous variable in the multivariate regression models.

**Table 1:** General maternal and child characteristics according to children’s T-score categories of CPRS-R:S subscales at age 7 years.

	T-score of CPRS-R:S subscales, n (%)							
	Oppositional		Cognitive problems/inattention		Hyperactivity		ADHD Index	
	< 65	≥ 65	< 65	≥ 65	< 65	≥ 65	< 65	≥ 65
	n = 1112 (92.4)	n = 92 (7.6)	n = 1057 (87.8)	n = 147 (12.2)	n = 1035 (86)	n = 169 (14)	n = 1085 (90.4)	n = 115 (9.6)
***Maternal characteristics***								
Age (years), mean (SD)	30.8 (3.9)	30.5 (3.6)	**30.9 (3.9)**	**29.8 (4.3)†**	**30.9 (3.9)**	30.2 (4.0)*	**30.9 (3.9)**	**29.6 (4.3)†**
Pre-pregnancy BMI (kg/m^2^), mean (SD)	23.4 (4.0)	24.0 (4.7)	**23.3 (4.0)**	24.2 (4.4)*	**23.3 (3.9)**	24.1 (5.1)*	**23.3 (4.0)**	**24.7 (4.6)†**
**Ethnic group, n (%)**								
Caucasian	1097 (98.6)	90 (97.8)	1042 (98.6)	145 (98.6)	1020 (98.5)	167 (98.8)	1075 (98.7)	112 (97.4)
Other	15 (1.3)	2 (2.2)	15 (1.4)	2 (1.4)	15 (1.5)	2 (1.2)	14 (1.3)	3 (2.6)
**Educational level, n (%)**								
Primary or less	**226 (20.3)**	**28 (30.4)**	**207 (19.7)**	**47 (32.0)**	**207 (20.0)**	**47 (27.8)**	**219 (20.1)**	**35 (30.4)**
Secondary	**451 (40.6)**	**39 (42.4)**	**433 (40.9)**	**57 (38.8)**	**418 (40.4)**	**72 (42.6)**	**437 (40.1)**	**53 (46.1)**
University	**435 (39.1)**	25 (27.2)*	**417 (39.4)**	**43 (29.2)†**	**410 (39.6)**	50 (29.6)*	**433 (39.8)**	**27 (23.5)†**
**Social class, n (%)**								
Low	638 (58.4)	59 (64.1)	**595 (56.3)**	**102 (69.4)**	**583 (56.5)**	**112 (66.3)**	**612 (56.2)**	**85 (73.9)**
Medium	200 (18.0)	15 (16.3)	**190 (18.0)**	**25 (17.0)**	**183 (17.7)**	**32 (18.9)**	**199 (18.3)**	**16 (13.9)**
High	274 (24.6)	18 (19.6)	**272 (25.7)**	**20 (13.6)†**	**267 (25.8)**	**25 (15.8)†**	**278 (25.5)**	**14 (12.2)†**
**Smoking during pregnancy, n (%)**								
Yes	**304 (27.3)**	36 (39.1)*	**289 (27.3)**	51 (34.7)*	**280 (27.1)**	60 (35.5)*	**292 (26.8)**	**48 (41.7)†**
No	**808 (72.7)**	**56 (61.9)**	**768 (72.7)**	**96 (65.3)**	**755 (72.9)**	**109 (65.5)**	**797 (73.2)**	**67 (58.3)**
Alcohol intake during pregnancy (g/d), mean (SD)	0.31 (0.9)	0.28 (0.7)	0.32 (1.0)	0.21 (0.5)	0.29 (0.9)	0.38 (1.1)	0.31 (0.9)	0.26 (0.7)
**Mode of delivery, n (%)**								
Eutocic	690 (62.0)	56 (60.9)	660 (62.4)	86 (58.5)	647 (62.5)	99 (58.6)	680 (62.4)	66 (58.4)
Dystocic	422 (38.0)	36 (39.1)	397 (37.6)	61 (41.5)	388 (37.5)	70 (41.4)	409 (37.6)	49 (42.6)
Parity, n (%)								
Primiparous	629 (56.6)	51 (55.4)	591 (55.9)	89 (60.5)	579 (55.9)	101 (59.8)	**605 (55.5)**	**75 (65.2)**
Multiparous	483 (43.4)	41 (44.6)	466 (44.1)	58 (39.5)	456 (44.1)	68 (40.2)	**484 (44.5)**	40 (34.8)*
Breastfeeding (months), mean (SD)	27.0 (19.8)	26.2 (21.4)	**27.5 (19.8)**	**22.9 (20.2)†**	**27.7 (19.9)**	**21.8 (19.8)†**	**27.6 (19.9)**	**20.6 (19.6)†**
***Iron characteristics and other parameters***								
**Haemoglobin (g/dL), mean (SD)**								
1st trimester	12.9 (0.8)	12.8 (0.8)	12.9 (0.8)	12.8 (0.9)	12.9 (0.9)	12.8 (0.9)	12.8 (0.8)	12.9 (0.9)
3rd trimester	11.6 (0.9)	11.5 (1.0)	11.6 (0.9)	11.5 (1.0)	11.6 (0.9)	11.5 (1.0)	11.6 (0.9)	11.6 (0.9)
**Anaemia (Hb < 110 g/L), n (%)**								
1st trimester	20 (2.0)	2 (2.4)	18 (1.9)	4 (3.0)	17 (1.8)	5 (3.3)	19 (1.9)	3 (2.9)
3rd trimester	268 (25.6)	25 (29.8)	253 (25.5)	40 (29.2)	249 (25.7)	44 (27.3)	264 (25.8)	29 (27.4)
Ferritin levels (µg/L) at 1st period, median (IQR)^€^	29.0 (17.8, 45.9)	26.8 (15.5, 42.0)	28.7 (17.0, 45.5)	30.9 (20.0, 46.4)	28.9 (17.0, 45.8)	29.0 (20.0, 44.4)	28.6 (17.0, 45.2)	31.1 (20.7, 47.8)
**Iron deficiency at 1st trimester, n (%)**								
Yes	139 (12.5)	10 (10.9)	138 (13.1)	11 (7.5)	135 (13.0)	14 (8.2)	141 (13.0)	8 (7.0)
No	973 (87.5)	82 (89.1)	919 (86.9)	136 (92.5)	900 (86.9)	155 (91.7)	948 (87.0)	107 (93.0)
**Iron supplementation, n (%)**								
Yes	584 (52.5)	56 (60.9)	553 (52.3)	87 (59.2)	543 (52.5)	97 (57.4)	572 (52.5)	68 (59.1)
No	528 (47.5)	36 (39.1)	504 (47.7)	60 (40.8)	492 (47.5)	72 (42.6)	517 (47.5)	47 (40.9)
Total iron intake (mg/d), mean (SD)	29.3 (27.3)	32.9 (33.5)	29.8 (28.2)	27.6 (24.9)	29.6 (28.3)	28.9 (24.9)	29.6 (27.8)	29.1 (27.9)
**Total iron intake ≥ 27 mg/d, n (%)**								
Yes	370 (33.2)	37 (40.2)	355 (33.6)	52 (35.4)	347 (33.5)	60 (35.5)	370 (34.0)	37 (32.2)
No	742 (66.7)	55 (59.8)	702 (66.4)	95 (64.6)	688 (66.5)	109 (64.5)	719 (66.0)	78 (67.8)
**CRP ≥ 0.65 mg/L at 1st trimester, n (%)⁋**								
Yes	182 (26.4)	20 (33.3)	180 (27.0)	22 (27.2)	175 (26.8)	27 (28.1)	**176 (25.9)**	26 (37.7)*
No	507 (73.6)	40 (66.7)	488 (73.0)	59 (72.8)	478 (73.2)	69 (71.9)	**504 (74.1)**	**43 (62.3)**
***Child characteristics***								
**Children’s sex, n (%)**								
Female	**571 (51.3)**	**26 (28.3)**	**513 (48.5)**	**84 (57.1)**	518 (50.1)	79 (46.7)	548 (50.3)	49 (42.6)
Male	**541 (48.6)**	**66 (71.7)†**	**544 (51.5)**	63 (42.9)*	517 (49.9)	90 (53.3)	541 (49.7)	66 (57.4)
Age at delivery (weeks), mean (SD)	**39.8 (1.2)**	40.1 (1.2)*	**39.8 (1.2)**	40.1 (1.3)*	39.8 (1.2)	39.9 (1.2)	**39.8 (1.2)**	40.1 (1.3)*
Birth weight (g), mean (SD)	3336 (384)	3309 (444)	3333 (386)	3344 (408)	**3347 (385)**	**3256 (403)†**	3333 (382)	3348 (449)
Birth head circumference (cm), mean (SD)	34.5 (1.2)	34.3 (1.2)	34.5 (1.2)	34.4 (1.3)	**34.5 (1.2)**	34.2 (1.2)*	34.5 (1.2)	34.4 (1.3)
Age at time of test (years), mean (SD)	7.3 (0.5)	7.3 (0.5)	7.3 (0.5)	7.3 (0.5)	**7.3 (0.5)**	7.2 (0.5)*	7.3 (0.5)	7.3 (0.5)
BMI-for-age z-score at 7 years, mean (SD)	0.80 (1.2)	0.71 (1.4)	0.77 (1.2)	0.90 (1.3)	0.80 (1.2)	0.71 (1.3)	0.77 (1.2)	0.90 (1.4)

Values are expressed in mean (SD) or number (%) unless otherwise indicated. The differences between child T-scores of CPRS-R:S subscales as derived from Student’s t or chi-square tests, as appropriate.

*SD* standard deviation, *BMI* body mass index, *IQR* interquartile range, *CPRS-R:S* Conners’ Parent Rating Scale-Revised Short Form, *ADHD* attention deficit hyperactivity disorder.

Bold values denote a significant statistical test; *p-value < 0.05 or †p-value < 0.01. ^€^FS measurements at first and second trimester as a whole were defined as first period of pregnancy. ⁋n = 683.

Lastly, we also performed separate multivariate logistic regression analyses to assess the risk (ORs, 95% CIs) of elevated T-scores (≥ 65) (as a dichotomous variable indicating the presence or absence of ADHD symptoms) for each CPRS-R:S subscale in 7-year-old children associated with maternal total log-SF levels (per SD increment) in the first period of pregnancy (first and second trimester combined) and trimester-specific Hb levels (first and third trimester).

In all analyses, a two-sided *p* < 0.05 was considered significant. The data was analysed using the application STATA, version 15.0 (StataCorp LP, College Station, TX, USA).

### Ethical approval

All procedures performed in the study were in accordance with the ethical standards of Donostia (Gipuzkoa), La Fe (Valencia) Hospitals, and the Medical Assistance Municipal Institute (Barcelona) and with the 1964 Helsinki declaration and its later amendments or comparable ethical standards.

## Results

The study sample consisted of 1204 mother–child (49% girls) pairs with complete data on the main variables of interest, maternal SF and child CPRS-R:S. The mean age of the mothers was 30.8 years (SD 3.9 years) and mean pre-pregnancy BMI was 23.5 kg/m^2^ (SD 4.1 kg/m^2^). Most of the women (98.6%) were Caucasians, 38.2% had secondary/university studies, 24.2% were of high social class, and 21% smoked during pregnancy. Median maternal SF during the first period of pregnancy (first and second trimester combined) was 28.9 µg/L (inter quartile range (IQR) 17.5–45.5 μg/L). Average Hb during the first and third trimester was 12.8 (SD 0.8 g/L) and 11.6 g/L (SD 0.9 g/L), respectively. Of the 1,204 women, only 2% were anaemic (Hb < 11.0 g/dL; n = 22) in the first trimester and 12% (n = 149) had iron deficiency (SF < 12 μg/L) in the first period of pregnancy. As expected, the proportion of women with anaemia increased (26%; n = 293) in the third trimester of pregnancy.

The proportion of children with elevated T-scores (≥ 65) for each of the CPRS-R:S subscales was 7.6% for Oppositional, 12.2% for Cognitive problems/Inattention, 14% for Hyperactivity, and 9.6% for the ADHD index (Table 1). Overall, children of younger mothers, with higher BMIs or who reported smoking during pregnancy were more likely to present elevated T-scores (≥ 65) on the CPRS-R:S subscales. Conversely, ADHD symptoms were less frequent in the children of mothers with higher social and educational classes, and in those who breastfed longer (except for the Oppositional subscale). There was no difference in child ADHD-related outcomes in terms of maternal anaemia or iron status.

The women of the cohort included in this analysis were older, had a higher level of education and social class, and tended to be smokers more than non-participating women (n = 946; all *p* < 0.05, data not shown). The general characteristics for women in each cohort are shown in Supplementary Table 1 and highlight the diversity of the different cohorts.

In the univariate linear regression analysis, maternal total log-SF was positively associated with the children’s T-scores on the CPRS-R:S subscales Cognitive problems/Inattention (β: 0.63, 95%CI: 0.06–1.19; *p* = 0.029) and ADHD index (β: 0.72, 95%CI: 0.20–1.24; *p* = 0.007). These associations were not present after adjusting for cohort region, mother’s age, pre-pregnancy self-reported BMI, social class, smoking during pregnancy, total iron intake above the RDA (27 mg/d), mode of delivery, parity, breastfeeding duration, children’s sex, age at delivery, birth weight, birth head circumference, children’s age at the time of the test, and BMI-for-age z-score at 7 years of age. We also added Hb levels during the first trimester of pregnancy to the model in order to evaluate the independent impact of SF levels on outcomes and vice versa. Similarly, no association was observed in the analyses stratified by first and second trimester of pregnancy (Table 2). Hb levels in the first and third trimester of pregnancy were not related to the children’s T-scores in any of the CPRS-R:S subscales in univariate or multivariate-adjusted models (Table 2). Moreover, additional CRP adjustment, which was available only in 683 observations (56.7%), did not substantially alter the estimates (data not shown).

**Table 2:** Unadjusted and multivariate-adjusted linear regression models of the associations between maternal SF and Hb levels (continuous, tertiles) and children’s T-score of CPRS-R:S subscales at age 7 years.

	T-score of CPRS-R:S subscales							
	Oppositional		Cognitive Problems/Inattention		Hyperactivity		ADHD Index	
	β (95% CI)	*p* value	β (95% CI)	*p* value	β (95% CI)	*p* value	β (95% CI)	*p* value
SF levels (µg/L) at 1st period of gestation (n = 1204)*								
Log-SF (per SD, continuous), unadjusted model	0.15 (− 0.41, 0.70)	0.60	**0.63 (0.06, 1.19)**	**0.029**	0.37 (− 0.21, 0.95)	0.21	**0.72 (0.20, 1.24)**	**0.007**
Log-SF (per SD, continuous), multivariate-adjusted model†	0.33 (− 0.24, 0.91)	0.25	0.47 (− 0.11, 1.05)	0.11	0.26 (− 0.34, 0.85)	0.40	0.50 (− 0.04, 1.04)	0.08
	R^2^ = 0.043, adj.R^2^ = 0.026, F (19, 1081) = 2.56, p < 0.001		R^2^ = 0.076, adj.R^2^ = 0.060, F (19, 1081) = 4.70, p < 0.001		R^2^ = 0.077, adj.R^2^ = 0.061, F (19, 1081) = 4.74, p < 0.001		R^2^ = 0.079, adj.R^2^ = 0.063, F (19, 1081) = 4.90, p < 0.001	
T1 (< 21.5 μg/L)	0 (Ref.)		0 (Ref.)		0 (Ref.)		0 (Ref.)	
T2 (21.5–38 μg/L)	0.51 (− 0.86, 1.88)	0.46	0.21 (− 1.17, 1.59)	0.77	0.94 (− 0.47, 2.35)	0.19	0.29 (− 1.00, 1.57)	0.66
T3 (≥ 38 μg/L)	0.18 (− 1.20, 1.57)	0.79	0.40 (− 1.00, 1.83)	0.57	− 0.09 (− 1.51, 1.33)	0.90	0.46 (− 0.84, 1.76)	0.49
*p* for trend^€^	0.89		0.58		0.69		0.50	
	R^2^ = 0.042, adj.R^2^ = 0.025, F (20, 1080) = 2.39, p < 0.001		R^2^ = 0.074, adj.R^2^ = 0.057, F (20, 1080) = 4.34, p < 0.001		R^2^ = 0.078, adj.R^2^ = 0.061, F (20, 1080) = 4.60, p < 0.001		R^2^ = 0.077, adj.R^2^ = 0.060, F (20, 1080) = 4.50, p < 0.001	
**SF levels (µg/L) at 1st trimester (n = 633)**								
Log-SF ((per SD, continuous), multivariate-adjusted model†	− 0.11 (− 0.94, 0.71)	0.79	0.46 (− 0.38, 1.30)	0.28	− 0.08 (− 0.99, 0.84)	0.86	0.54 (− 0.24, 1.32)	0.17
	R^2^ = 0.053, adj.R^2^ = 0.021, F (19, 565) = 1.67, p = 0.037		R^2^ = 0.121, adj.R^2^ = 0.09, F (19, 565) = 4–09, p < 0.001		R^2^ = 0.079, adj.R^2^ = 0.050, F (19, 565) = 2.57, p < 0.001		R^2^ = 0.011, adj.R^2^ = 0.080, F (19, 565) = 3.67, p < 0.001	
T1 (< 24.2 μg/L)	0 (Ref.)		0 (Ref.)		0 (Ref.)		0 (Ref.)	
T2 (24.2–41 μg/L)	0.20 (− 1.70, 2.09)	0.84	0.24 (− 1.67, 2.16)	0.80	1.23 (− 0.85, 3.31)	0.35	0.97 (− 0.81, 2.745	0.29
T3 (≥ 41 μg/L)	− 0.84 (− 2.74, 1.05)	0.38	0.28 (− 1.63, 2.19)	0.77	− 1.31 (− 3.39, 0.77)	0.21	0.45 (− 1.33, 2.23)	0.62
*p* for trend^€^	0.32		0.79		0.12		0.75	
	R^2^ = 0.055, adj.R^2^ = 0.022, F (20, 564) = 1.65, p = 0.037		R^2^ = 0.121, adj.R^2^ = 0.09, F (20, 564) = 3.82, p < 0.001		R^2^ = 0.090, adj.R^2^ = 0.056, F (20, 564) = 2.57, p < 0.001		R^2^ = 0.011, adj.R^2^ = 0.077, F (20, 564) = 3.44, p < 0.001	
**SF levels (µg/L) at 2nd trimester (n = 571)**								
Log-SF (per SD), multivariate-adjusted model†	0.57 (− 0.26, 1.40)	0.17	0.50 (− 0.33, 1.32)	0.23	0.50 (− 0.27, 1.28)	0.20	0.43 (− 0.33, 1.20)	0.26
	R^2^ = 0.056, adj.R^2^ = 0.020, F (19, 496) = 1.54, p = 0.066		R^2^ = 0.066, adj.R^2^ = 0.030, F (19, 496) = 1.83, p = 0.017		R^2^ = 0.077, adj.R^2^ = 0.042, F (19, 496) = 2.18, p = 0.003		R^2^ = 0.067, adj.R^2^ = 0.031, F (19, 496) = 1.83, p = 0.014	
T1 (< 19.3 μg/L)	0 (Ref.)		0 (Ref.)		0 (Ref.)		0 (Ref.)	
T2 (19.3–35.7 μg/L)	0.20 (− 1.83, 2.22)	0.85	0.90 (− 1.11, 2.91)	0.38	0.84 (− 1.04, 2.73)	0.38	0.24 (− 1.62, 2.11)	0.80
T3 (≥ 35.7 μg/L)	0.95 (− 1.12, 3.03)	0.37	1.32 (− 0.74, 3.38)	0.21	1.56 (− 0.38, 3.49)	0.11	1.13 (− 0.79, 3.04)	0.25
*p* for trend^€^	0.34		0.24		0.12		0.23	
	R^2^ = 0.054, adj.R^2^ = 0.016, F (20, 495) = 1.41, p = 0.109		R^2^ = 0.066, adj.R^2^ = 0.028, F (20, 495) = 1.75, p = 0.023		R^2^ = 0.078, adj.R^2^ = 0.041, F (20, 495) = 2.11, p = 0.003		R^2^ = 0.068, adj.R^2^ = 0.030, F (20, 495) = 1.79, p = 0.019	
**Hb (g/dL) at 1st trimester (n = 1101)**								
Hb (g/dL, continuous), unadjusted model	− 0.34 (− 0.99, 0.32)	0.31	− 0.14 (− 0.81, 0.54)	0.69	− 0.24 (− 0.92, 0.45)	0.50	− 0.05 (− 0.67, 0.57)	0.87
Hb (g/dL, continuous), multivariate-adjusted model†	− 0.24 (− 0.89, 0.41)	0.48	− 0.16 (− 0.82, 0.50)	0.63	− 0.29 (− 0.96, 0.38)	0.39	− 0.07 (− 0.68, 0.54)	0.83
	R^2^ = 0.043, adj.R^2^ = 0.027, F (19, 1081) = 2.58, p < 0.001		R^2^ = 0.076, adj.R^2^ = 0.060, F (19, 1081) = 4.72, p < 0.001		R^2^ = 0.080, adj.R^2^ = 0.061, F (19, 1081) = 4.71, p < 0.001		R^2^ = 0.080, adj.R^2^ = 0.064, F (19, 1081) = 4.96, p < 0.001	
T1 (< 12.5 g/dL)	0 (Ref.)		0 (Ref.)		0 (Ref.)		0 (Ref.)	
T2 (12.5–13.3 g/dL)	− 0.73 (− 2.08, 0.61)	0.28	− 1.21 (− 2.56, 0.15)	0.08	− 1.13 (− 2.52, 0.26)	0.11	− 0.85 (− 2.11, 0.41)	0.19
T3 (≥ 13.3 g/dL)	− 0.49 (− 1.86, 0.88)	0.48	− 0.09 (− 1.47, 1.29)	0.90	− 0.24 (− 1.65, 1.17)	0.74	0.10 (− 1.18, 1.38)	0.88
*p* for trend^€^	0.48		0.90		0.72		0.86	
	R^2^ = 0.044, adj.R^2^ = 0.026, F (20, 1080) = 2.49, p < 0.001		R^2^ = 0.080, adj.R^2^ = 0.063, F (20, 1081) = 4.689, p < 0.001		R^2^ = 0.078, adj.R^2^ = 0.061, F (20, 1081) = 4.59, p < 0.001		R^2^ = 0.082, adj.R^2^ = 0.066, F (20, 1081) = 4.86, p < 0.001	
**Hb (g/dL) at 3rd trimester (n = 1129)**								
Hb (g/dL, continuous), unadjusted model	− 0.01 (− 0.62, 0.60)	0.96	− 0.30 (− 0.92, 0.32)	0.34	− 0.31 (− 0.95, 0.33)	0.34	− 0.27 (− 0.85, 0.30)	0.35
Hb (g/dL, continuous), multivariate-adjusted model†	− 0.06 (− 0.66, 0.55)	0.86	− 0.26 (− 0.86, 0.35)	0.40	− 0.29 (− 0.92, 0.33)	0.35	− 0.25 (− 0.81, 0.32)	0.39
	R^2^ = 0.041, adj.R^2^ = 0.025, F (19, 1109) = 2.50, p < 0.001		R^2^ = 0.077, adj.R^2^ = 0.061, F (19, 1109) = 4.88, p < 0.001		R^2^ = 0.073, adj.R^2^ = 0.058, F (19, 1109) = 4.62, p < 0.001		R^2^ = 0.078, adj.R^2^ = 0.062, F (19, 1109) = 4.91, p < 0.001	
T1 (< 11.1 g/dL)	0 (Ref.)		0 (Ref.)		0 (Ref.)		0 (Ref.)	
T2 (11.1–12 g/dL)	− 0.52 (− 1.86, 0.83)	0.45	− 0.34 (− 1.69, 1.01)	0.62	− 0.42 (− 1.81, 0.97)	0.55	− 0.21 (− 1.47, 1.04)	0.74
T3 (≥ 12 g/dL)	0.18 (− 1.16, 1.53)	0.79	− 0.10 (− 1.44, 1.25)	0.89	− 0.33 (− 1.72, 1.06)	0.64	− 0.18 (− 1.43, 1.08)	0.78
*p* for trend^€^	0.82		0.88		0.63		0.78	
	R^2^ = 0.042, adj.R^2^ = 0.025, F (20, 1108) = 2.43, p < 0.001		R^2^ = 0.077, adj.R^2^ = 0.060, F (20, 1108) = 4.61, p < 0.001		R^2^ = 0.073, adj.R^2^ = 0.056, F (20, 1108) = 4.36, p < 0.001		R^2^ = 0.077, adj.R^2^ = 0.060, F (20, 1108) = 4.63, p < 0.001	

*CI* confidence interval, *SD* standard deviation, *BMI* body mass index, *T* tertile, *SF* serum ferritin, *Hb* haemoglobin, *CPRS-R:S* Conners’ Parent Rating Scale-Revised Short Form, *ADHD* attention deficit hyperactivity disorder.

Linear regression models were used to calculate β coefficient (β) and 95% confidence intervals (CIs). Bold values denote a significant statistical test at a level of statistical significance p-value < 0.05. †Models were adjusted for cohorts (Gipuzkoa, Sabadell, Valencia), maternal age (years), maternal pre-pregnancy BMI (kg/m^2^), social class (low, medium, high), smoking during pregnancy (yes, no), mode of delivery (eutocic, dystocic), parity (primiparous, multiparous) breastfeeding (months), total iron intake above the RDA (27 mg/d) (yes, no), children’s sex (male, female), age at delivery (weeks), birth weight (kg), birth head circumference (cm), children’s age at time of test (years) and BMI-for-age z-score at age 7 years, log-SF levels in 1st period (μg/L) (for Hb analyses) and Hb levels (for SF analyses). ^€^Tests for trend were conducted using the median values for each tertile of SF or Hb as a continuous variable in the multivariate regression models. *SF measurements at first and second trimester as a whole were defined as first period of pregnancy.

**Figure 1:**
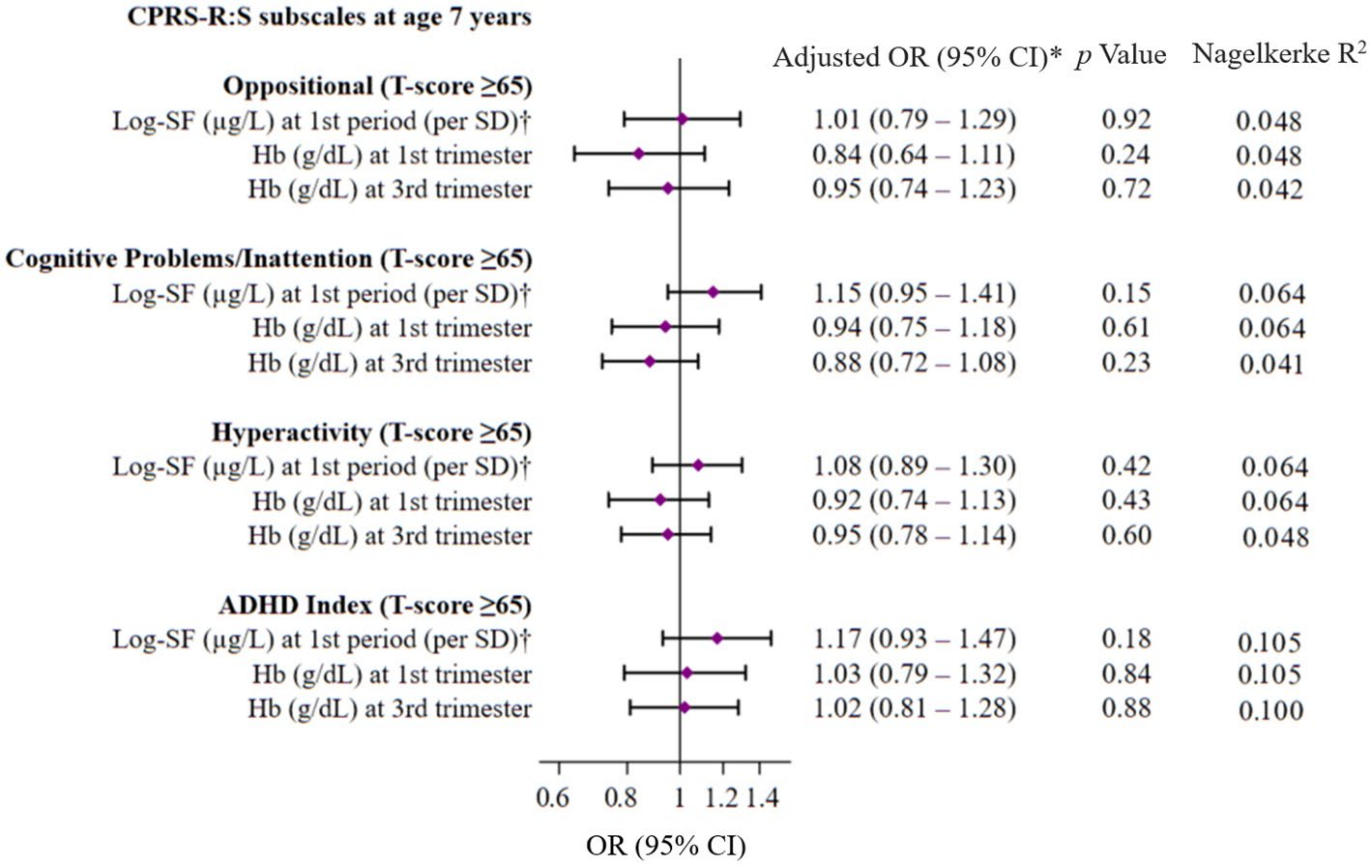
Multivariate-adjusted ORs (95%(CIs) of children’s T-score ≥ 65 for CPRS-R:S subscales at age 7 years associated with maternal log-SF and Hb levels (continuous). *Models were adjusted for cohorts (Gipuzkoa, Sabadell, Valencia), maternal age (years), maternal pre-pregnancy BMI (kg/m^2^), social class (low, medium, high), smoking during pregnancy (yes, no), mode of delivery (eutocic, dystocic), parity (primiparous, multiparous) breastfeeding (months), total iron intake above the RDA (27 mg/d) in 2^nd^ period (yes, no), children’s sex (male, female), age at delivery (weeks), birth weight (kg), birth head circumference (cm), children’s age at time of test (years) and BMI-for-age z-score at age 7 years, log-SF levels in 1^st^ period (μg/L) (for Hb analyses) and Hb levels (for SF analyses). †FS measurements at first and second trimester as a whole were defined as first period of pregnancy. The diamonds represent OR and the whisker plots represent 95% CIs. *BMI* body mass index, *SF* serum ferritin, *CPRS-R:S* Conners’ Parent Rating Scale-Revised Short Form, *ADHD* attention deficit hyperactivity disorder.

Figure 1 shows the multivariate-adjusted ORs (95%CIs) of the children’s T-score ≥ 65 for CPRS-R:S subscales at age 7 years associated with maternal total log-SF levels in the first period of pregnancy (first and second trimester combined) and trimester-specific Hb levels**.** We consistently observed no association between maternal SF and Hb levels and the risk of ADHD symptomatology among school-aged children. In addition, the estimates remained the same regardless of the pregnancy trimester when the SF measurements were taken (data not shown). We observed no effects of the children’s sex on interactions (all p for interaction > 0.05).

## Discussion

The present study investigated the associations between maternal SF and Hb levels during pregnancy and ADHD symptoms in 7-year-old children from the Spanish cohorts of the INMA project. We observed no relationship between these indicators of iron status (SF and Hb) and ADHD symptoms.

In terms of iron parameters, a large body of evidence suggests that ADHD is related to lower SF levels in children^15,16,29–31^. However, the body of evidence regarding maternal SF and Hb levels and their effect on childhood ADHD is much more limited, consisting of only two previous studies^7,21^ and the present data, which means that the true extent of any such effects remains unresolved. Our findings here regarding SF levels were relatively inconsistent with our previous findings from the same INMA cohort at age 4 years^7^. At 4 years old, we reported a significant inverse association between maternal SF levels during pregnancy and Inattentive-type and total ADHD symptom scores in boys, but positive associations in girls. However, no associations were found with Hyperactivity-type symptom scores. This apparent disagreement between the two assessment periods, at 4 and 7 years of age, may be partly explained by the age differences of the children since ADHD symptoms alter over time as children grow^32,33^. Also, positive environmental influences (e.g. school, family, nutrition, etc.) on the brain development of children may have impaired or nullified any effect of maternal ferritin on ADHD symptoms in children at school age. Likewise, other child factors such as SF levels or pharmacological and/or psychological interventions at 4 and 7 years old will also probably influence their neurocognitive abilities. This information is not available now, but it is worth examining in future research. Finally, methodological differences between the two INMA studies should be considered. That is, the instrument that we used to measure the ADHD symptoms (the CPRS-R:S at 7 years old) in the present analysis was different from the previous INMA study (the ADHD Rating Scale-IV at 4 years old) which was completed by the children’s classroom teacher and not by parents. This could decrease the comparability of results between both age periods because agreement between parents and teachers regarding ADHD symptoms is usually relatively poor^34^. It also limits our ability to disentangle age from both instrument and informant influence on the ADHD symptoms score. Likewise, in the present study, standardized T-scores enabled us to identify the presence and/or absence of ADHD symptoms from the cut-off-scores (i.e., T-score ≥ 65) so the approach was more clinical.

As far as the children’s sex is concerned, in our study, the overall prevalence rate of ADHD symptoms (T-score ≥ 65) was 9.3%, and was slightly higher in boys (10.9%) than in girls (8.2%), which is in line with previous studies^2^. We also found sex-related differences in specific symptom profiles, which may be largely due to biological factors related to brain sex differences (e.g. hormones and genes)^35,36^; specifically, boys had higher Oppositional and Hyperactivity Symptom scores than girls. However, in contrast with data from the INMA study at 4 years old, we found no evidence for sex differences in the associations between maternal SF levels during pregnancy and children’s T-scores in any of the CPRS-R:S subscales or ADHD symptomatology at 7 years of age (all interaction terms, p value > 0.05).

In addition to assessing the iron deposited by ferritin, the present study also examined the relation between maternal Hb concentrations during pregnancy, a marker of iron metabolism used to detect the presence of anaemia with or without iron deficiency, and ADHD in offspring. There was no significant association between maternal Hb levels and anaemia (based on Hb levels < 11.0 g/dL) and any child ADHD symptoms. In this context, Wiegersma et al.^21^ recently found that maternal anaemia diagnosed before but not after 30 weeks of gestation in a large cohort of 299,768 Swedish women and their 532,232 offspring aged 6–29 was associated with increased offspring risk of ADHD, which co-occurred with autism and/or intellectual disability disorder. Nonetheless, although this association is partly consistent with our findings, it was not statistically significant when only ADHD was considered as the diagnosis. In this regard, to check critical periods of susceptibility, we examined whether the associations between levels of maternal Hb (in the first and third trimester) and SF (in the first and second trimester) and child CPRS-R:S scores might differ depending on the trimester of pregnancy. However, unlike previous studies on child neurodevelopment^13^, our work consistently yielded null results for ADHD symptoms. It should be noted that our study provided information about SF and Hb levels at different points in pregnancy, so our findings might reflect representative exposure throughout pregnancy. Furthermore, no previous studies have evaluated both indicators of iron status throughout pregnancy.

The present study also supported other socio-environmental factors during pregnancy that have been consistently linked to ADHD in offspring, such as lower maternal education and social class^37^, giving birth at younger ages^38^, high pre-pregnancy BMI^37^, smoking^39,40^, and breastfeeding duration^41^. Importantly, in our analyses we controlled for all these factors in addition to other potential confounders or mediators linked to ADHD in the literature, and we found that the associations between maternal SF or Hb levels during pregnancy and the development of ADHD symptoms in children remained unchanged. Taken together, the paucity of available evidence and the inconsistency of findings in this field of research make it difficult to draw any firm conclusions about the causal relationship between maternal SF and Hb levels during pregnancy and ADHD symptoms at school age. Further research is required to confirm/refute this tentative hypothesis.

The present study has several strengths. First of all, the sample size is relatively large, which increases the reliability of any conclusions that are drawn from it. Furthermore, we examined all ADHD symptoms both as a continuous outcome (score) and as a dichotomous outcome (symptom diagnostic criteria) to ensure a more clinical approach. Our analyses were also controlled for a wide variety of modifiable prenatal and postnatal factors related to the risk of ADHD symptoms. Nevertheless, we also acknowledge some limitations. Firstly, the study assessed ADHD symptomatology but not diagnosis with standardized criteria. Thus, the CPRS-R:S collects symptoms from the last month but does not assess duration and severity, the age of onset, or interference in child functioning. In addition, although the Conners’ Parent Rating Scale has shown good psychometric properties in Spanish school populations and is reliable and valid for detecting ADHD^42–44^, we obtained information from only one source —the family and not the school. It may be important that ADHD symptoms be perceived by different observers (i.e., parent and teacher) because these symptoms are required to be present in multiple settings. Moreover, other factors in children such as ferritin levels, intellectual quotient, psychological or pharmacological treatments and familial risk of ADHD, which has been linked to child ADHD symptoms, were not collected. Finally, sample sizes for trimester-specific associations regarding FS were limited (first trimester, n = 633, and third trimester, n = 571), so we cannot discard the possibility that they are underpowered. Thus, this issue merits further research.

In summary, this study provides no evidence to support any relationship between maternal iron status during pregnancy and symptom severity or risk of ADHD symptoms in 7-year-old children. Nevertheless, these findings should be replicated with the use of fully standardized ADHD diagnostic instruments during different assessment periods and for longer follow-up times if we are to understand the trajectory patterns of this complex outcome. Randomized trials are also required to determine how different doses of iron supplementation during pregnancy adapted to initial levels of SF and Hb can affect ADHD diagnosis in offspring.

## Supplementary Information

Supplementary Information.

## Data Availability

The datasets generated and/or analyzed during the current study are not publicly available due to subject confidentiality but are available from the corresponding author on reasonable request.
