# Author Correction: Maternal iron status during pregnancy and attention deficit/hyperactivity disorder symptoms in 7-year-old children: a prospective cohort study

**DOI:** 10.1038/s41598-023-27876-x

**Published:** 2023-01-18

**Authors:** Andrés Díaz‑López, Josefa Canals-Sans, Jordi Julvez, Silvia Fernandez-Barrés, Sabrina Llop, Marisa Rebagliato, Nerea Lertxundi, Loreto Santa‑Marina, Mònica Guxens, Jordi Sunyer, Victoria Arija

**Affiliations:** 1 Nutrition and Mental Health Research Group (NUTRISAM), Rovira I Virgili University (URV), C/ Sant Llorenç 21, 43201 Reus, Tarragona Spain; 2 Institut d’Investigació Sanitària Pere Virgili (IISPV), Tarragona, Spain; 3 Research Center for Behavioral Assessment (CRAMC), Rovira I Virgili University (URV), Tarragona, Spain; 4 Barcelona Institute for Global Health (ISGlobal), Barcelona, Spain; 5 Epidemiology and Environmental Health Joint Research Unit, FISABIO-Public Health, FISABIO-Universitat Jaume I-Universitat de València, Valencia, Spain; 6 Spanish Consortium for Research On Epidemiology and Public Health (CIBERESP), Instituto de Salud Carlos III, Madrid, Spain; 7 Biodonostia, Epidemiology and Public Health Area, Environmental Epidemiology and Child Development Group, San Sebastian, Spain; 8 Faculty of Psychology, University of the Basque Country (UPV/EHU), San Sebastian, Spain; 9 Pompeu Fabra University, Barcelona, Spain; 10 Department of Child and Adolescent Psychiatry/Psychology, Erasmus MC, University Medical Centre, Rotterdam, The Netherlands

Correction to: *Scientific Reports* 10.1038/s41598-022-23432-1, published online 01 December 2022

The original version of this Article contained errors in the spelling of the authors Josefa Canals-Sans and Silvia Fernandez-Barrés which were incorrectly given as Josefa Canals Sans and Silvia Fernandez-Bares.

In addition, affiliation 1 contained an error, which was incorrectly given as ‘Department of Basic Medical Sciences, Nutrition and Mental Health Research Group (NUTRISAM), Rovira I Virgili University (URV), C/ Sant Llorenç 21, 43,201 Reus, Tarragona, Spain’. The correct affiliation is listed below:

Nutrition and Mental Health Research Group (NUTRISAM), Rovira I Virgili University (URV), C/ Sant Llorenç 21, 43,201, Reus, Tarragona, Spain.

The original Article has been corrected.

