# Supplementary material for: Maternal iron status during pregnancy and attention deficit/hyperactivity disorder symptoms in 7-year-old children: a prospective cohort study
Source: Sci Rep. 2022 Dec 1;12:20762. doi: 10.1038/s41598-022-23432-1 (PMC9715623; doi:10.1038/s41598-022-23432-1)
Supplement: Supplementary file 1 — Supplementary Information. [file 41598_2022_23432_MOESM1_ESM.docx]

**Supplementary Table 1.** General characteristics of the INMA cohort study by geographic area

| **Characteristics** | | **Gipuzkoa** | **Sabadell** | **Valencia** | ***p* value** |
| --- | --- | --- | --- | --- | --- |
|  |  | **N=381** | **N=455** | **N=368** |  |
| Maternal age (years), mean (SD) | | 31.4 (3.2) | 30.6 (4.23) | 30.4 (4.1) | **0.002** |
| Maternal pre-pregnancy BMI (kg/m^2^), mean (SD) | | 22.9 (3.4) | 23.7 (4.5) | 23.7 (4.1) | **0.010** |
| Ethnic group, n (%) | |  |  |  |  |
|  | Caucasian | 377 (99.5) | 445 (98.0) | 362 (93.4) | 0.19 |
|  | Other | 2 (0.5) | 9 (1.8) | 6 (1.6) |  |
| Maternal educational level, n (%) | |  |  |  |  |
|  | Primary or less | 39 (10.2) | 111 (24.4) | 104 (28.3) |  |
|  | Secondary | 137 (36.0) | 195 (42.9) | 158 (42.9) | **<0.001** |
|  | University | 2045(53.8) | 149 (32.7) | 106 (28.8) |  |
| Maternal social class, n (%) | |  |  |  |  |
|  | Low | 219 (57.5) | 244 (53.6) | 234 (63.6) |  |
|  | Medium | 54 (14.2) | 92 (20.2) | 69 (18.8) | **0.001** |
|  | High | 108 (28.3) | 119 (26.2) | 65 (17.6) |  |
| Smoking during pregnancy, n (%) | |  |  |  |  |
|  | Yes | 81 (21.3) | 124 (27.2) | 135 (36.7) | **<0.001** |
|  | No | 300 (78.7) | 331 (72.8) | 233 (63.3) |  |
| Alcohol intake during pregnancy (g/d), mean (SD) | | 0.19 (0.46) | 0.34 (1.04) | 0.39 (1.09) | **0.007** |
| Mode of delivery, n (%) | |  |  |  |  |
|  | Eutocic | 258 (67.7) | 302 (66.4) | 186 (50.5) | **<0.001** |
|  | Dystocic | 123 (32.3) | 153 (33.6) | 182 (49.5) |  |
| Parity, n (%) | |  |  |  |  |
|  | Primiparous | 215 (56.4) | 256 (56.3) | 209 (56.8) | 0.99 |
|  | Multiparous | 166 (43.6) | 199 (43.7) | 159 (43.2) |  |

Values are expressed in mean (SD) or number (%). *p*-values for the differences between geographic areas as derived from ANOVA or chi-square tests, as appropriate. Abbreviations: BMI, body mass index; and SD, standard deviation. The significance of numbers in bold is p-value<0.05
